# Integrating metagenomics, transcriptomics, and molecular docking to reveal core gene biomarkers and gut microbiota regulatory mechanisms in tuberculous meningitis

**DOI:** 10.3389/fimmu.2026.1844914

**Published:** 2026-09-09

**Authors:** Dan Cui, Xinxin Ren, Na Li

**Affiliations:** Department of Tuberculosis, Hebei Chest Hospital, Shijiazhuang, Hebei, China

**Keywords:** bile acid metabolites, gut microbiota, Janus kinase 2 (JAK2), phosphatidylinositol-3-kinase catalytic subunit beta (PIK3CB), tuberculous meningitis

## Abstract

**Purpose:**

Tuberculous meningitis (TBM) is a severe extrapulmonary tuberculosis with high mortality and neurological sequelae, while the role of gut microbiota and its metabolites in TBM pathogenesis remains poorly understood. This study aimed to characterize gut microbiota alterations in TBM patients and elucidate the “microbiota-metabolite-gene” regulatory axis.

**Methods:**

Fecal samples from 11 TBM patients and 11 healthy controls were subjected to 16S rRNA sequencing. Core target genes were identified via differential expression screening, machine learning, immune infiltration analysis and gene set enrichment analysis based on the public GSE40586 dataset. The regulatory axis was constructed by database prediction and molecular docking, and the regulatory effect was verified by *in vitro* functional assays in THP-1-derived macrophages.

**Results:**

TBM patients exhibited significant gut dysbiosis. Two core genes (PIK3CB and JAK2) were identified, which were positively correlated with pro-inflammatory immune cells and enriched in bacterial infection pathways. The constructed regulatory axis showed that upregulated gut bacteria produced bile acid metabolites targeting PIK3CB/JAK2, with strong binding affinity verified by molecular docking. *In vitro* experiments verified that CDCA dose-dependently upregulated PIK3CB and JAK2 expression and promoted pro-inflammatory activation of macrophages, while silencing of target genes significantly reversed this effect.

**Conclusion:**

This study identifies a “gut microbiota-bile acid-PIK3CB/JAK2” regulatory axis in TBM, thus providing novel insights into gut-brain crosstalk and potential diagnostic biomarkers and therapeutic targets.

## Introduction

Tuberculosis (TB) is a major infectious disease worldwide, with high rates of morbidity and mortality, particularly in developing countries (1). Tuberculous meningitis (TBM), the most lethal form of extrapulmonary tuberculosis, accounts for 10–15% of all extrapulmonary cases and is associated with poor neurological outcomes (2). *Mycobacterium tuberculosis* disseminates hematogenously to the meninges and brain parenchyma, forming multiple tuberculous granulomas in the subcortical region. The rupture of these granulomas releases bacilli into the cerebrospinal fluid (CSF), leading to TBM (3). TBM is characterized by meningeal inflammation and a thick gelatinous exudate at the base of the brain. Cerebral infarction, a common complication of TBM, affects up to two-thirds of patients and contributes to poor prognosis (4).

The gut-brain axis has been hypothesized to be a key mechanism by which the gut microbiota can influence brain health. The gut microbiota modulates host neuronal function by producing various metabolites, including peptides, neurotransmitters, and neuroactive compounds (5). Gut dysbiosis can disrupt the intestinal barrier, subsequently triggering intestinal inflammation and peripheral inflammatory responses. This, in turn, can compromise blood-brain barrier (BBB) functioning, induce central nervous system inflammation, and can lead to hypothalamic-pituitary-adrenal axis dysfunction, ultimately triggering neurological dysfunction (6). Prior studies have found marked changes in gut microbiome profiles when comparing patients with meningitis with healthy controls (7). However, the potential pathogenic role of gut microbial communities in meningitis pathogenesis has not yet been conclusively established.

Although prior studies have reported alterations in gut microbiota composition in patients with TBM, the specific roles of the different gut microbial communities and their metabolites in TBM pathogenesis remain poorly elucidated. Through the production of metabolites, the gut microbiota not only participates in maintaining local intestinal immune homeostasis but also influences the immune response and inflammatory reactions in the central nervous system via the “gut-brain axis.” However, systematic studies linking gut microbiota alterations, associated metabolites, and host central immune gene expression regulation in patients with TBM are currently lacking.

This study used the following analyses to determine the role of the gut microbiota in TBM: (1) 16S rRNA sequencing to analyze differences in gut microbiota between patients with TBM and healthy controls; (2) analysis of the gutMGene database to mine for metabolites associated with differential microbiota; (3) analysis of the SuperPred database to predict metabolite targets; and (4) integration of these targets with transcriptomic data from the GSE40586 dataset derived from TBM.

By applying machine learning algorithms to screen core genes combined with immune infiltration analysis, single-gene GSEA enrichment analysis, and molecular docking validation, the potential molecular mechanisms by which gut microbiota metabolites can regulate host gene expression were systematically explored. The final aim of this study was to reveal the role of the “gut microbiota-metabolite-immune gene axis” in TBM pathogenesis, providing new ideas and a theoretical basis for the identification of diagnostic markers and new therapeutic targets for TBM.

## Materials and methods

### Study subjects and sample collection

Eleven patients with TBM (TBM group) and 11 healthy controls (HC group) were enrolled in this study. This study was approved by the Ethics Committee of Hebei Chest Hospital (Ethics Approval No.: 2023099), and all participants provided written informed consent to participate.

Fresh fecal samples were collected from all participants, immediately placed in sterile cryovials, and stored at -80 °C until DNA extraction.

The inclusion criteria for the TBM group were: ① met the clinical diagnostic criteria for TBM (based on cerebrospinal fluid examination, imaging features, and clinical manifestations); ② age ≥ 18 years; ③ no history of anti-tuberculosis treatment or having received treatment for < 72 hours. The exclusion criteria were: ① concurrent diagnosis with other central nervous system infections; ② any malignancy, autoimmune disease, or severe hepatic or renal insufficiency; ③ use of antibiotics, probiotics, or immunosuppressants within the past month; ④ pregnant or lactating women.

The inclusion criteria for the HC group were: ① healthy individuals undergoing concurrent health check-ups, without any neurological symptoms or signs of infection; ② age and sex matched with the TBM group; ③ no history of tuberculosis or tuberculosis exposure. The exclusion criteria were the same as the TBM group.

### RT-qPCR validation of core gene expression

Peripheral venous blood samples were collected from 11 patients with tuberculous meningitis and 11 healthy controls. Each sample is repeated three times. Peripheral blood mononuclear cells were isolated, and total RNA was extracted using TRIzol reagent (Cat. No. 15596026, Invitrogen, USA). RNA integrity was assessed by agarose gel electrophoresis, and RNA concentration and purity were determined using a NanoDrop 2000 spectrophotometer (Thermo Fisher Scientific, USA). A total of 1 μg of RNA was reverse transcribed into cDNA using the PrimeScript™ RT Reagent Kit (Cat. No. RR037A, Takara, Japan) under the following conditions: 37 °C for 15 min, followed by 85 °C for 5 s. Subsequently, real-time quantitative PCR was performed using the SYBR^®^ Premix Ex Taq™ II Kit (Cat. No. RR820A, Takara, Japan) on an Applied Biosystems 7500 Real-Time PCR System (Thermo Fisher Scientific, USA). The reaction mixture was 20 μL, and the amplification protocol consisted of initial denaturation at 95 °C for 30 s, followed by 40 cycles of 95 °C for 5 s and 60 °C for 34 s. GAPDH (Cat. No. HQP006001, Sangon Biotech, China) was used as an internal reference gene. The relative expression levels of PIK3CB and JAK2 mRNA were calculated using the 2^−ΔΔCt method. All experiments were performed in triplicate. Differences between the two groups were analyzed using an independent samples t-test, with P < 0.05 considered statistically significant.

### Fecal DNA extraction and 16S rRNA gene sequencing

Total DNA was extracted from 200 mg of fecal samples using a specific DNA extraction kit (DP328, TIANGEN BIOTECH CO., LTD, China). DNA integrity was further assessed by agarose gel electrophoresis, and the concentration and purity were determined using a NanoDrop 2000 (Thermo Fisher Scientific, China). The V3-V4 hypervariable region of the bacterial 16S rRNA gene was selected for PCR amplification using primers 338F (5’-ACTCCTACGGGAGGCAGCAG-3’) and 806R (5’-GGACTACHVGGGTWTCTAAT-3’). PCR products were recovered and purified through 2% agarose gel electrophoresis, after which they were subjected to paired-end sequencing on the Illumina Nextseq2000 platform. Sequencing was conducted by Majorbio Bio-Pharm Technology Co., Ltd.

### 16S sequencing analysis of gut microbiota changes in patients with TBM

Our 16S rRNA sequencing was outsourced to Meiji Biotechnology Co., Ltd. and conducted using the Meiji Bioinformatics Cloud Platform. We performed DNA extraction on clinical samples, designed and synthesized primers and adapters, carried out PCR amplification and product quantification and normalization, and prepared the library using the NEXTFLEX Rapid DNA-Seq Kit, followed by sequencing.

Raw sequencing reads were quality-controlled using the fastp software (https://github.com/OpenGene/fastp, version 0.23.0), and assembly was performed using the FLASH software (https://ccb.jhu.edu/software/FLASH/, version 1.2.11): bases with quality scores below 20 at the end of reads were filtered out, and a 50-bp window was set. Paired reads were merged into a single sequence with a minimum overlap length of 10 bp. The USEARCH software (http://drive5.com/usearch/, version 11) was used to cluster sequences into OTUs based on 97% similarity, following this specific workflow: non-repeated sequences were extracted from the optimized sequences, and single sequences without repeats were removed. Cluster the non-duplicate sequences into OTUs based on 97% similarity; remove chimeras during the clustering process to obtain representative OTU sequences. Map all optimized sequences to the representative OTU sequences, select sequences with a similarity of 97% or higher to the representative OTU sequences, generate an OTU table, and remove sequences annotated as chloroplast and mitochondrial from all samples. The number of sequences per sample was normalized to 20,000, and the RDP classifier (http://rdp.cme.msu.edu/, version 11.5) against the Silva 16S rRNA gene database (v138.2) to perform OTU taxonomic annotation, with a confidence threshold of 70%, and the community composition of each sample was analyzed at different taxonomic levels. PICRUSt2 (version 2.2.0) software was used to perform 16S functional prediction analysis.

The 22 fecal samples were sequenced and analysis. The composition and abundance distribution of the gut microbiota across different taxonomic levels were analyzed using the Sobs, Shannon, and Simpson indices for assessment. R software (version 4.6.1) was used to perform principal component analysis (PCA) and principal coordinate analysis (PCoA), and to analyze microbial community composition at the genus/phylum levels. PCA is based on Euclidean distance, while PCoA is based on Bray-Curtis distance. Combined with PERMANOVA permutation test. Using the `stats` package in R (version 4.6.1) and the `scipy` package in Python (v1.0.0), we performed a Wilcoxon signed-rank test (two-tailed, P < 0.05) to analyze differences in the genus-level microbial community composition between the two groups.

OTU analysis primarily involved the clustering and transforming of sequencing reads (sequences) and alignment with species databases to obtain taxonomic information and abundance of microorganisms in clinical samples. Rank-abundance curves were constructed to assess species abundance and evenness in the samples, while pan-core species analysis was applied to evaluate whether the sequencing sample size was sufficient. This method is a classic 16S RNA analysis method, which can stably achieve genus-level classification annotation and meet the requirements for differentiating microbial communities in this study. The subsequent analysis was based on the tables generated by this process.

Raw sequencing data were spliced using FLASH (v1.2.11) (8), with quality control conducted using Trimmomatic (v0.33) (9). OTUs were clustered at 97% similarity using UCLUST (v1.2.22) (10). Representative sequences of OTUs were annotated against the Silva (v138) database (11), with a confidence threshold ≥ 0.7. All alpha diversity indices, including the Sobs (observed OTUs), Shannon, and Simpson indices, were calculated using Mothur (v1.30.2) (12). Rarefaction curves were plotted to assess the sequencing depth. Venn diagrams were constructed based on OTU distribution to display the shared and unique OTUs between the two groups. PCA and PCoA were performed using the vegan package in R, while the Beta diversity was assessed based on Bray-Curtis distance.

### Species composition and differential analysis

The relative abundance of different species at the phylum and genus levels was calculated for each sample, and community composition bar plots were subsequently generated using R to visualize the microbial community structures of the two patient groups. To compare species abundance differences between groups, the Wilcoxon rank-sum test (two-tailed, P < 0.05) was applied to analyze the differentially abundant species at the genus level, with the Benjamini-Hochberg method applied to correct any P-values for multiple comparisons. The obtained results are preliminary differences at the relative abundance level.

### Differential microbiota analysis

To identify the differentially abundant microbiota between patients with TBM and healthy controls, the raw species abundance data was first preprocessed. The fold change (FC) and adjusted P-value (P_adjust) were used as criteria to screen differential microbiota. The fold change was calculated as the ratio of the mean abundances in the TBM and HC groups. The screening criteria were as follows: first, the log2-transformed fold change (log_2_FC) was calculated; |log_2_FC| values > 0.585 and < 0 indicated up- and down-regulation of the microbiota in the TBM group, respectively. The significance threshold was set at |log_2_FC| > 0.585 (equivalent to an original fold change > 1.5), and adjusted P-value < 0.05 (13, 14). Microbiota were classified into three categories, based on these thresholds: significantly upregulated (log_2_FC > 0.585 and P_adjust < 0.05), significantly downregulated (log_2_FC < -0.585 and P_adjust < 0.05), and non-significant. To further display any key differential microbiota, the five microbiota with the highest and lowest log_2_FC values among all significantly up- and down-regulated taxa, respectively, were selected as representative species. Subsequently, significantly upregulated differential microbiota for further analysis were selected.

### Metabolite information retrieval based on the gutMGene database

To explore the potential functions of differential microbiota, the gutMGene database (http://bio-computing.hrbmu.edu.cn/gutmgene) was used to retrieve information regarding the metabolites associated with significantly upregulated microbiota. This database, developed by the team at Harbin Medical University, contains host protein-coding genes that have been experimentally confirmed to be involved in maintaining intestinal barrier function, regulating gut microbiota–host interactions, mediating enteric inflammatory responses, and modulating metabolic homeostasis. For each microbiota, the related metabolite information, including the metabolite name, sample type, and references were extracted. All search results were manually verified and compiled for subsequent analysis.

### Metabolite target prediction and core target screening

To predict the targets of metabolites associated with differential microbiota, the two-dimensional structure information of metabolites were first obtained from the PubChem database (https://pubchem.ncbi.nlm.nih.gov/) and subsequently converted it to the SMILES format. Subsequently, target prediction was conducted using the SuperPred database (https://prediction.charite.de). This database integrates the compound-target interaction data from SuperTarget, ChEMBL, and BindingDB, excluding any interactions with weak binding (Ki, IC50 > 10 μM); it contains approximately 341,000 compounds, 1,800 targets, and 665,000 interaction records. Metabolite names or SMILES structures were input into the SuperPred database, which uses ECFP molecular fingerprints to calculate the structural similarity and outputs predicted targets with similarity scores. After obtaining predicted targets for all metabolites associated with upregulated microbiota, the intersections among the targets for each metabolite were used as core targets for subsequent analysis.

### GSE40586 dataset processing and analysis

To validate the expression of core targets in TBM, GSE40586 dataset, which contains 18 normal samples and 21 meningitis samples, was extracted from the GEO database (https://www.ncbi.nlm.nih.gov). The GEOquery package was used to read the expression matrix and clinical information. Samples were divided into the Normal and BM groups based on sample source. Probe IDs were converted to gene symbols using the platform annotation file; for cases where multiple probes corresponded to the same gene, the average value was taken as the expression value of that gene. Differential expression analysis was conducted using the limma package, with linear model fitting and empirical Bayes methods employed to identify differentially expressed genes (DEGs). The false discovery rate (FDR) was controlled using the Benjamini-Hochberg method. The screening criteria were updated to |log_2_FC| > 0.585 and FDR < 0.05. A total of 1228 differentially expressed genes were identified. The screening threshold was set at |log_2_FC| > 0.585 and P < 0.05, classifying genes into upregulated, downregulated, and non-significant categories. Finally, gene symbols were converted to ENTREZ IDs using the clusterProfiler package for subsequent analysis.

### Intersection gene screening and functional enrichment analysis

To identify the key genes associated with the gut microbiota, the intersection of the DEGs and gut-related genes obtained from ID mapping files were used to generate an intersection gene set. A Venn diagram was drawn using the VennDiagram package to visualize the overlap between the two gene sets. Functional enrichment analysis was subsequently conducted on the intersection genes. Using the clusterProfiler package and org.Hs.eg.db database, gene symbols were converted to ENTREZ IDs. GO (Gene Ontology) and KEGG (Kyoto Encyclopedia of Genes and Genomes) enrichment analyses were subsequently conducted using all detected genes as the background set. GO analysis covered three categories: Biological Process (BP), Cellular Component (CC), and Molecular Function (MF). KEGG analysis was applied to identify associated signaling pathways. The Benjamini-Hochberg method was applied for multiple testing correction, with a screening criterion of adjusted P-value < 0.05.

### PPI network construction and core gene screening

To further screen for key genes, protein-protein interaction (PPI) networks were constructed using GeneMANIA and Cytoscape software. Intersecting genes were imported into the GeneMANIA database (http://genemania.org) to obtain gene interaction relationships and construct a relevant network. Simultaneously, an interaction network based on PPI data from the STRING database (https://string-db.org) was constructed using Cytoscape software. In Cytoscape, the cytoHubba plugin was used for topological analysis of the network, employing five different algorithms to calculate the node importance: Degree, EPC (Edge Percolated Component), MCC (Maximal Clique Centrality), MNC (Maximum Neighborhood Component), and Radiality. The top 10 core genes from each algorithm were subsequently selected. The intersections of the core genes obtained by the five algorithms were taken as the final set of the differential core genes for subsequent analysis.

### Machine learning-based core gene screening and validation

To further identify key diagnostic markers from the core genes screened by the PPI network, two machine learning algorithms, LASSO regression and random forest, were comprehensively applied for feature selection. The expression data of the intersecting core genes shared by the five algorithms (Degree, EPC, MCC, MNC, and Radiality) were used as the input features, with sample grouping (BM vs Normal) used as the response variable. First, LASSO regression analysis was conducted. A binomial logistic regression model was subsequently constructed using the glmnet package, and the optimal penalty parameter λ (lambda.1se) was determined by 5-fold cross-validation to select the feature genes with non-zero coefficients. Meanwhile, a random forest model was constructed using the randomForest package, with 500 decision trees. Feature importance was assessed based on the Gini coefficient (Mean Decrease Gini), and genes with importance above the median were selected. The intersection of genes selected by LASSO and random forest was taken as the final set of the key feature genes. A 5-fold cross-validation strategy was employed to evaluate the stability and diagnostic performance of the selected genes. All data were randomly divided into 5 parts, using 4 and 1 parts as the training and test sets, respectively, in rotation. The random forest model was trained iteratively, and the prediction accuracy, precision, recall, and F1 score were calculated. Receiver operating characteristic (ROC) curves were plotted, and the area under the curve (AUC) was calculated to evaluate the model’s discriminative ability. Finally, SHapley Additive exPlanations (SHAP) was applied for interpretability analysis of the final model. The fastshap package was used to calculate SHAP values for each feature per sample, quantifying the contribution of each gene to the prediction outcome. SHAP feature importance bar plots and SHAP scatter plots were generated to display the importance ranking of each gene and its relationship with the prediction outcome.

### Core gene validation

To validate the expression of the screened core genes (PIK3CB and JAK2) in meningitis and their correlation with immune cells, the following analyses were conducted: First, the expression data of PIK3CB and JAK2 in the Normal and BM groups were extracted. A faceted violin plot, combined with boxplot and scatter points, was subsequently drawn using the ggplot2 package to display expression distribution between the two groups. The Wilcoxon test was applied to assess the significance of intergroup differences. Pearson correlation was used to evaluate the correlation between PIK3CB and JAK2 expression, separately calculating the correlation coefficients for the overall sample, Normal group, and BM group. Scatter plots with linear regression lines were drawn using ggplot2 to display the correlation patterns. Subsequently, immune infiltration analysis was conducted. The ssGSEA method implemented in the GSVA package was used to calculate the enrichment scores for different immune cell types in samples based on immune cell marker gene sets. The Wilcoxon test was applied to identify immune cells with significant differences between groups (P < 0.05), while a combined boxplot-violin plot was drawn using ggplot2 to display the distribution of differential immune cells between the two groups. Finally, Pearson correlation coefficients between core genes and the enrichment scores of differential immune cells were calculated to evaluate the relationship between gene expression and immune infiltration levels.

### Single-gene GSEA analysis

To explore the potential biological functions of the identified core genes in TBM, single-gene GSEA analysis was conducted based on gene expression correlation. First, the Spearman correlation coefficients between the core genes and all other genes were calculated to identify gene sets significantly correlated with core gene expression. Subsequently, ene symbols were converted to ENTREZ IDs using the bitr function in the clusterProfiler package, and a gene list was constructed using the correlation coefficients as the ranking metric. Subsequently, KEGG pathway enrichment analysis was conducted using the gseKEGG function. The normalized enrichment score (NES) was applied to evaluate the enrichment degree of each pathway. Ridge plots were generated using the ridgeplot function to display gene distribution in enriched pathways, and bar plots were generated using ggbarplot to show the top 10 and bottom 10 pathways by NES, with colors mapped to adjusted P-values.

### Molecular docking

Molecular docking studies were performed to investigate the potential interaction mechanisms between the core genes and these key targets. The 3D structures of the target proteins were obtained from the Protein Data Bank (PDB) (https://www.rcsb.org), with priority given to experimentally determined, high-resolution structures with good structural completeness. For targets without experimental structures, 3D models were predicted using AlphaFold or similar algorithms to supplement the structural information. Prior to molecular docking, the protein receptor structures were uniformly preprocessed using AutoDock Tools 1.5.7, including the removal of water molecules, the addition of hydrogen atoms, the assignment of Gasteiger charges, and the definition of binding sites. The two-dimensional structures of different metabolites were obtained from PubChem and energy-minimized in Chem3D to obtain stable three-dimensional conformations. Molecular docking was performed using AutoDock Tools 1.5.7. During the docking process, the mesh box was set to fully enclose the entire protein molecule to ensure unbiased sampling across all potential binding sites. For each metabolite–core gene docking system, 50 independent docking experiments were performed, and the conformation with the lowest binding energy was selected as the optimal binding mode. Intermolecular interactions (such as hydrogen bonds, hydrophobic interactions, and π–π stacking) were further analyzed. Finally, by comprehensively comparing binding energy scores and interaction patterns, the affinity and specificity of the metabolites for each key target were evaluated, thereby elucidating their potential multi-target mechanisms of action from a structural perspective.

### Cell culture and drug treatment

The human monocyte line THP-1 was cultured in RPMI 1640 complete medium containing 10% fetal bovine serum in an incubator at 37 °C, 5% CO_2_, and saturated humidity. The cells were treated with 100 ng/mL phorbol methyl ester (PMA) for 24 hours to induce differentiation into adherent M0-type macrophages. Five concentration gradients of choline deoxycholate (CDCA) (HY-76847, MCE, USA) ranging from 0 to 200 μmol/L were established. After 24 hours of drug treatment, the cell culture supernatant and cell pellets were collected separately for subsequent analysis. The procedure was performed according to the instructions for the Lipofectamine 3000 transfection reagent. Specific siRNAs targeting PIK3CB and JAK2, as well as the negative control siNC, were mixed separately with the transfection reagent and added to the cell culture medium. Twenty-four hours after transfection, the culture medium was replaced with fresh medium containing the drug, and the efficiency of gene silencing was verified by Western blot. Perform the assays according to the instructions for the IL-6 (E-EL-H6156, Elabscience, China) and TNF-α (PT518, Beyotime, China) ELISA kits.

### Western blot analysis

Total proteins were extracted from cells in each group and quantified using the BCA method. Equal volumes of protein were subjected to SDS-PAGE electrophoresis and subsequently transferred to a PVDF membrane. After blocking with 5% skim milk at room temperature for 1 hour, the corresponding primary antibodies were added and incubated overnight at 4 °C. The primary antibodies used were as follows: PIK3CB (Affinity, DF2626, 1:1000), JAK2 (Affinity, AF6022, 1:1000), p-JAK2 (Affinity, AF3024, 1:1000), and GAPDH (HUABIO, ET1601-4, 1:50000). After washing the membrane three times with TBST, HRP-labeled secondary antibody (HUABIO, HA1001, 1:50000) was added and incubated at room temperature for 1 h. Development was performed using ECL chemiluminescent reagents, and the grayscale values of the bands were quantified using ImageJ software. Relative expression levels of total proteins were calculated using GAPDH as an internal control, and phosphoprotein levels were normalized relative to the corresponding total protein levels.

### Statistical analysis

Statistical analysis was primarily performed using R (version 4.6.1) and Python (scipy v1.0.0), with support from the Meiji Bioinformatics Cloud Platform (https://www.majorbio.com/). The complete list of R packages used in this study and their corresponding versions is as follows:

GEOquery (v2.78.0), stringr (v1.6.0), dplyr (v1.2.1), ggthemes (v5.2.0), limma (v3.66.0), sva (v3.58.0), ggplot2 (v4.0.3), factoextra (v2.1.0).

The Sobs, Shannon, and Simpson indices were used to assess species abundance and diversity. Differences in indices between groups were tested using the Wilcoxon rank-sum test and two-tailed tests. Beta diversity was analyzed using R; PCA was based on Euclidean distance, PCoA on Bray-Curtis distance, and combined with PERMANOVA permutation tests. Venn diagrams were analyzed using R. Abundance differences at the phylum and genus levels were analyzed using R. Tests for differences between groups were performed using the Wilcoxon signed-rank test, combined with Benjamini-Hochberg correction for multiple comparisons. For differential community analysis, the significance threshold was set at |log_2_FC| > 0.585 and P < 0.05. Differential gene expression in the GSE40586 dataset was analyzed using the limma package (|log_2_FC| > 0.585, P < 0.05). The validation of core genes was performed by assessing their correlations using the Wilcoxon test and Pearson correlation.

## Results

### 16S sequencing analysis of gut microbiota changes in patients with TBM

To verify the saturation and analytical reliability of the 16S rRNA sequencing data, we plotted dilution curves for the number of observed species (Sobs) and the Shannon index, respectively. As shown in **Supplementary Figures 1A, B**, as the number of sequenced reads increased, the dilution curves for all samples exhibited a trend of rapid initial rise followed by a gradual flattening, eventually reaching a stable plateau. This indicates that the sequencing depth in this study sufficiently covered the vast majority of microbial taxa within the samples, that the sequencing data volume was adequate, and that the subsequent diversity analysis results were stable and reliable. We used the Wilcoxon rank-sum test to compare alpha diversity between groups. The results showed statistically significant differences in gut microbiota diversity between patients with tuberculous meningitis (TBM) and healthy controls (HC). Specifically, both the Sobs index and the Shannon index in the TBM group were significantly lower than those in the HC group (**Supplementary Figures 1C, D**), suggesting a significant decline in species richness and an overall reduction in gut microbiota diversity among TBM patients; At the same time, the Simpson index in the TBM group was significantly higher than that in the HC group (**Supplementary Figure 1E**), reflecting a decrease in the evenness of species distribution in the gut microbiota of TBM patients, with the microbial community structure becoming concentrated around a few dominant species, exhibiting characteristics of structural disorganization.

**Figure 1:**
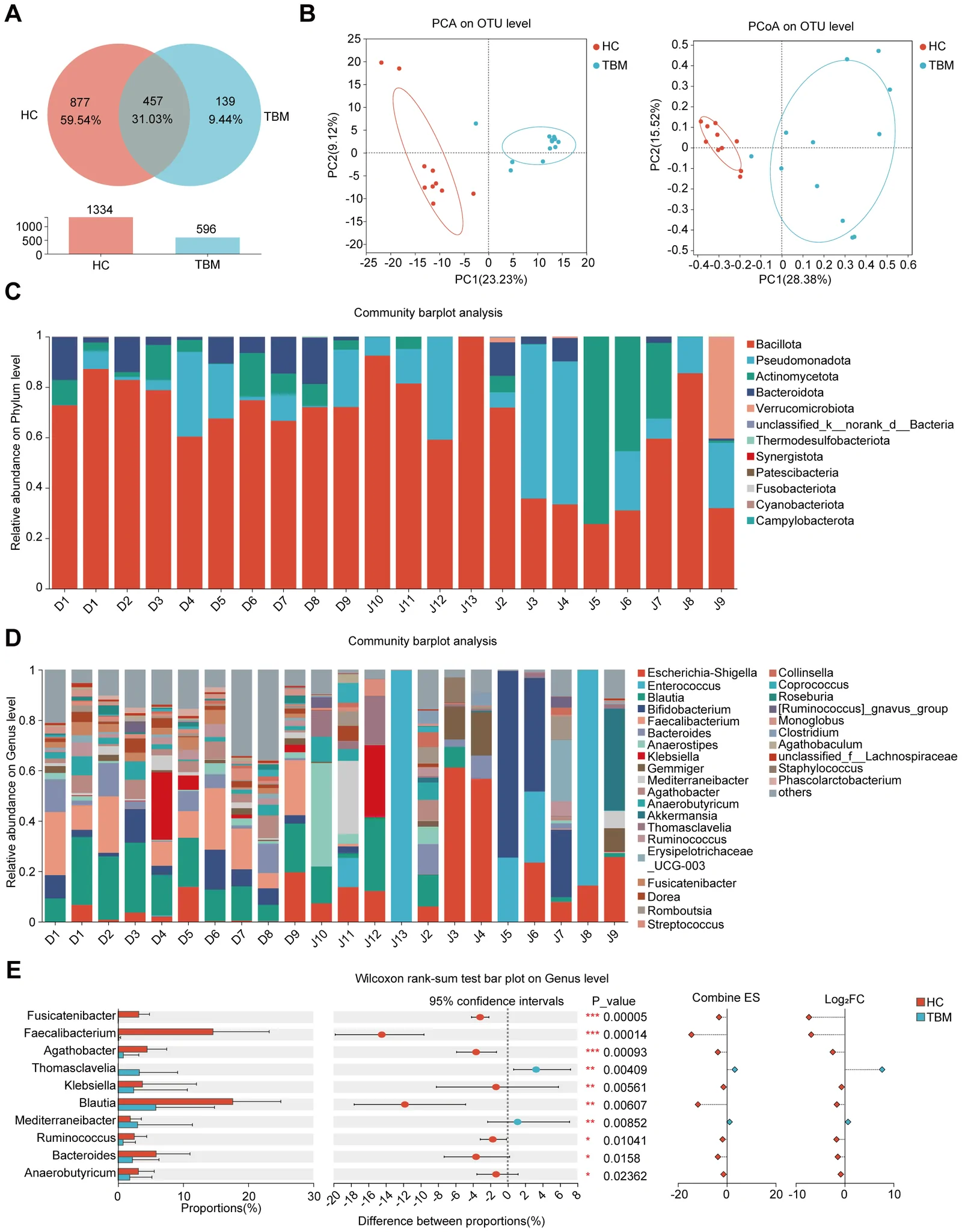
Analysis of gut microbiota changes in patients with tuberculous meningitis using 16S sequencing. **(A)** Venn diagram showing the numbers of shared and unique OTUs between the two groups, reflecting the degree of overlap in microbial composition. **(B)** Principal component analysis (PCA) based on OTU level, showing the differences in overall gut microbiota structure between the healthy control (HC) and tuberculous meningitis (TBM) groups. **(C)** Relative abundance distribution of species at the phylum level, comparing the dominant phyla and compositional differences between the HC and TBM groups. **(D)** Relative abundance distribution of species at the genus level, displaying the dominant genera and their relative abundance changes in the two groups. **(E)** The top 10 species with significant differences in relative abundance (ranked by P value) were screened using intergroup differential tests (e.g., LEfSe or Wilcoxon rank-sum test) to reveal potential marker microbiota associated with tuberculous meningitis.

PCA and PCoA revealed a clear separation in microbiota structure between the two groups, indicating significant differences in gut microbiota composition between the TBM and HC groups (**Figure 1A**). Further Venn diagram analysis of OTUs between the two groups identified 877 unique OTUs in the HC group and 139 unique OTUs in the TBM group, while the two groups shared 457 OTUs (**Figure 1B**).

At the phylum level, the relative abundances of species in the two groups were predominantly distributed among Bacillota, Pseudomonadota, and Actinomycetota (**Figure 1C**). At the genus level, the predominant genera included Escherichia-Shigella, Enterococcus, Blautia, Bifidobacterium, and Faecalibacterium (**Figure 1D**). Differential analysis further showed that the relative abundances of Fusicatenibacter and Faecalibacterium were significantly decreased in the TBM group compared with the HC group, while those of Thomasclavelia and Mediterraneibacter were significantly increased (**Figure 1E**).

### Differential gene analysis in tuberculous meningitis

**Figure 2:**
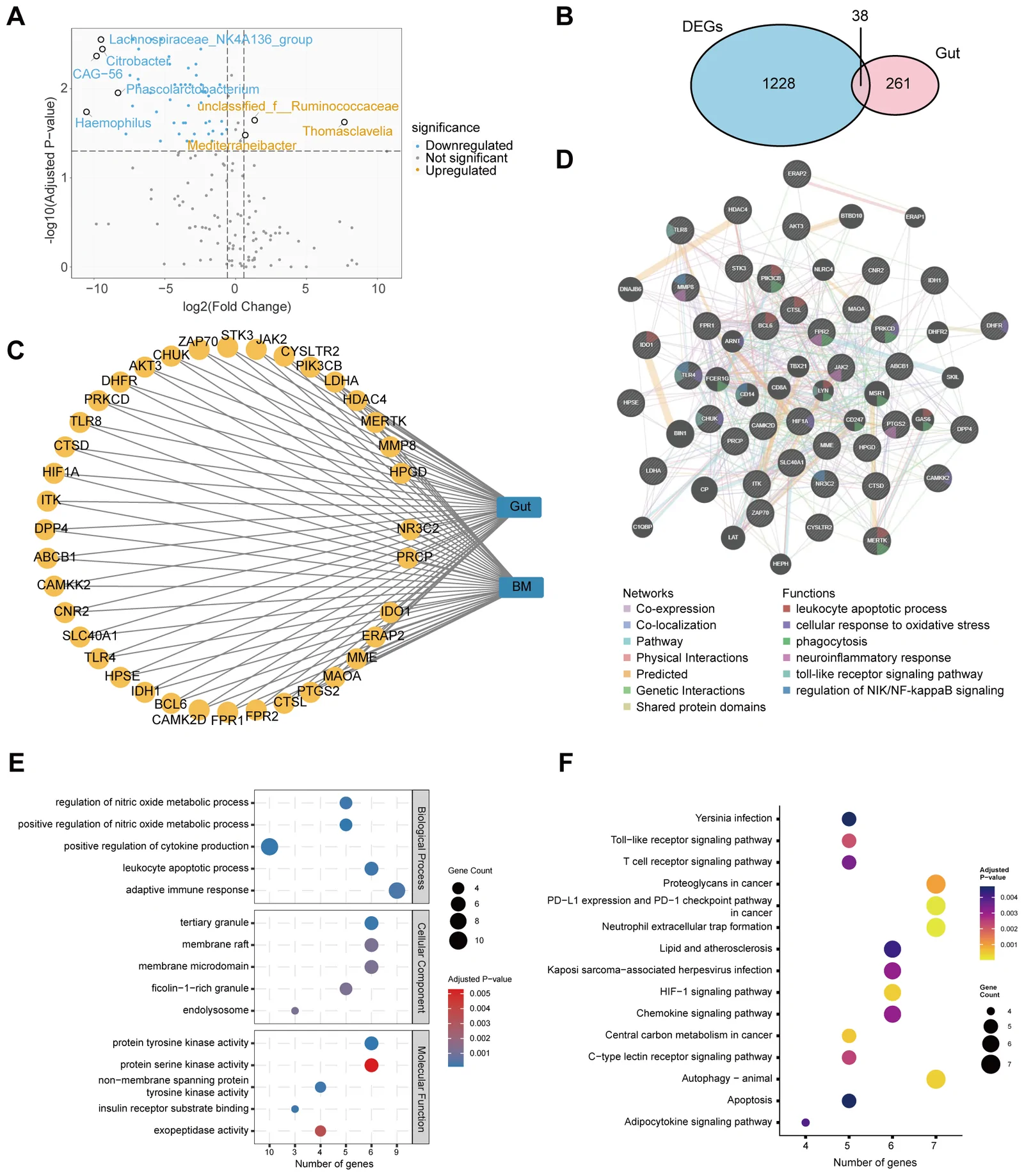
Differential gene analysis in tuberculous meningitis. **(A)** Volcano plot displaying the differential analysis results of microbial abundance between the TBM and HC groups. The screening criteria were |log_2_FC| > 0.585 (fold change > 1.5) and adjusted P < 0.05. Blue dots on the left represent significantly downregulated microbiota in the TBM group, while orange/yellow dots on the right represent significantly upregulated microbiota. **(B)** Venn diagram showing the intersection analysis between differentially expressed genes (DEGs) associated with meningitis screened from the GSE40586 dataset and a set of gut-related genes (299 genes). **(C)** Protein-protein interaction (PPI) network constructed using GeneMANIA. **(D)** Protein-protein interaction (PPI) network constructed using Cytoscape. **(E)** Gene Ontology (GO) functional enrichment analysis of the intersecting genes. **(F)** Kyoto Encyclopedia of Genes and Genomes (KEGG) pathway enrichment analysis of the intersecting genes.

To identify potential key genes linking gut microbiota to TBM pathogenesis, we first screened meningitis-related differentially expressed genes (DEGs) from the GSE40586 dataset, identifying a total of 1,266 DEGs. Concurrently, differential abundance analysis of gut microbiota between the TBM and healthy control groups was performed, and the results were visualized in a volcano plot (**Figure 2A**). Using predefined thresholds, three bacterial taxa—Thomasclavelia, unclassified f_Ruminococcaceae, and Mediterraneibacter—were found to be significantly enriched in the TBM group and were therefore selected for subsequent functional prediction and mechanistic investigation. To explore the potential interactions between gut microbiota and host gene expression, we then intersected the 1,286 DEGs with a curated set of 299 gut-related genes. This analysis yielded 38 overlapping genes (**Figure 2B**), representing candidate targets potentially regulated by gut microbiota in TBM; these were considered to be potential TBM-related targets regulated by the gut microbiota. To further reveal the interaction relationships among these intersection genes and to screen core genes, PPI networks were constructed using GeneMANIA and Cytoscape (**Figures 2C, D**). As shown in **Figure 2C**, the complex network interactions identified revealed the functional connections among these genes. To elucidate the biological functions of these 38 intersection genes, GO and KEGG enrichment analyses were conducted. GO enrichment analysis results (**Figure 2E**) showed that in terms of Biological Process (BP), the intersection genes were significantly enriched in immune-related terms, such as nitric oxide metabolic regulation, cytokine production, leukocyte apoptotic processes, and adaptive immune responses. Conversely, for Cellular Component (CC), they were mainly enriched in tertiary granules, membrane rafts, membrane microdomains, and lysosomes. For Molecular Function (MF), they were significantly enriched in protein serine/threonine kinase activity, non-membrane-spanning protein tyrosine kinase activity, and exopeptidase activity. KEGG pathway enrichment analysis (**Figure 2F**) showed that the intersection genes were primarily involved in multiple immune and inflammation-related pathways, including Yersinia infection, Toll-like receptor signaling pathway, T-cell receptor signaling pathway, PD-1/PD-L1 checkpoint pathway, neutrophil extracellular trap formation, HIF-1 signaling pathway, chemokine signaling pathway, autophagy, and apoptosis. These results indicate that the identified intersection genes are extensively involved in infection immunity, inflammatory responses, and cell death regulation. Functional annotation further revealed that the network was significantly enriched in immune-related processes, such as phagocytosis, T-cell receptor signaling, NF-κB signaling regulation, and neutrophil extracellular trap formation, corroborating the enrichment analysis results.

### Screening of key genes in TBM

**Figure 3:**
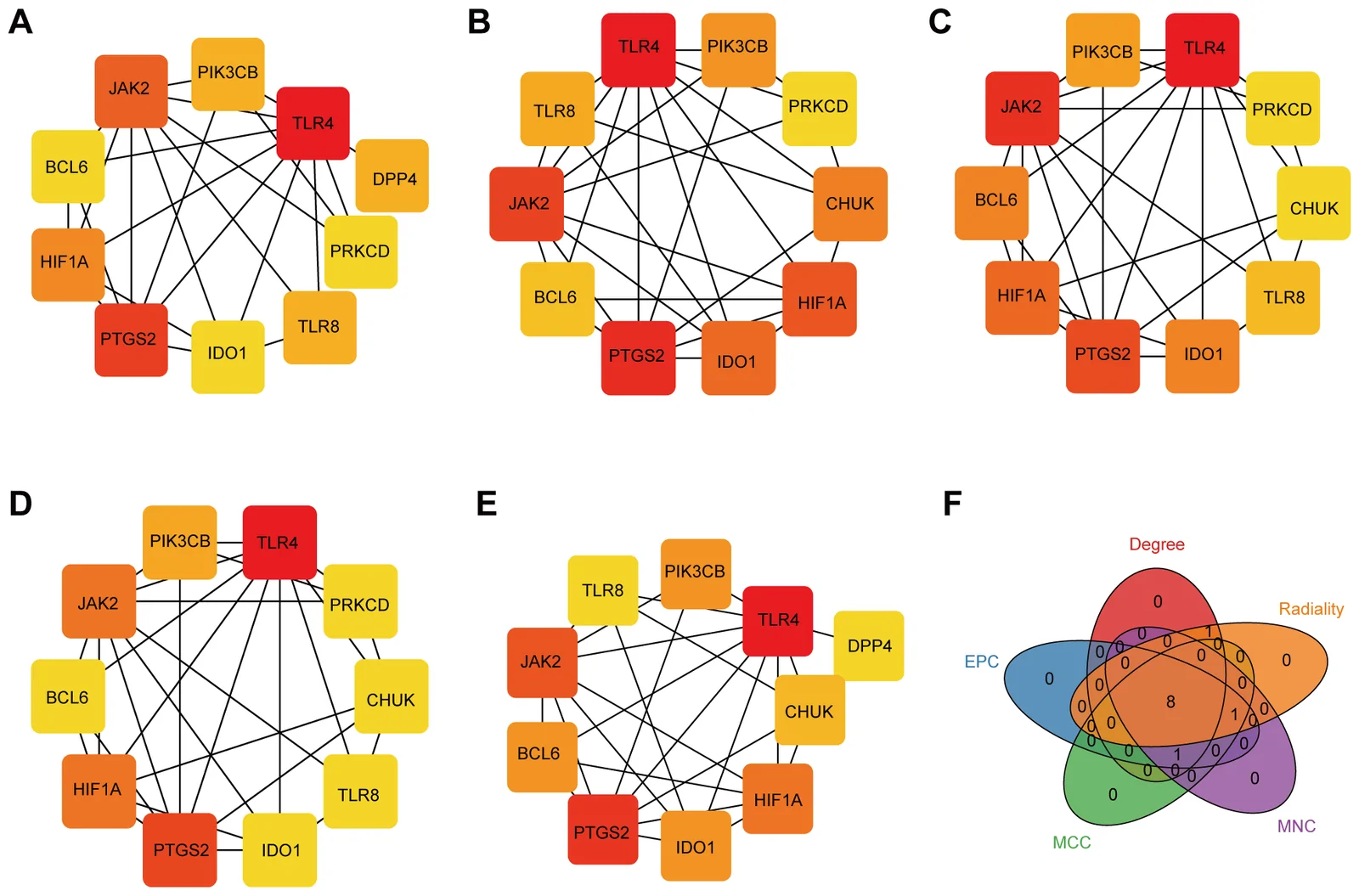
Screening of key genes in tuberculous meningitis. **(A–E)** Topological analysis of the network was performed using five different algorithms (Degree, EPC, MCC, MNC, and Radiality) implemented in the cytoHubba plugin. **(F)** Venn diagram showing the intersection of core genes obtained from the five algorithms.

**Figure 4:**
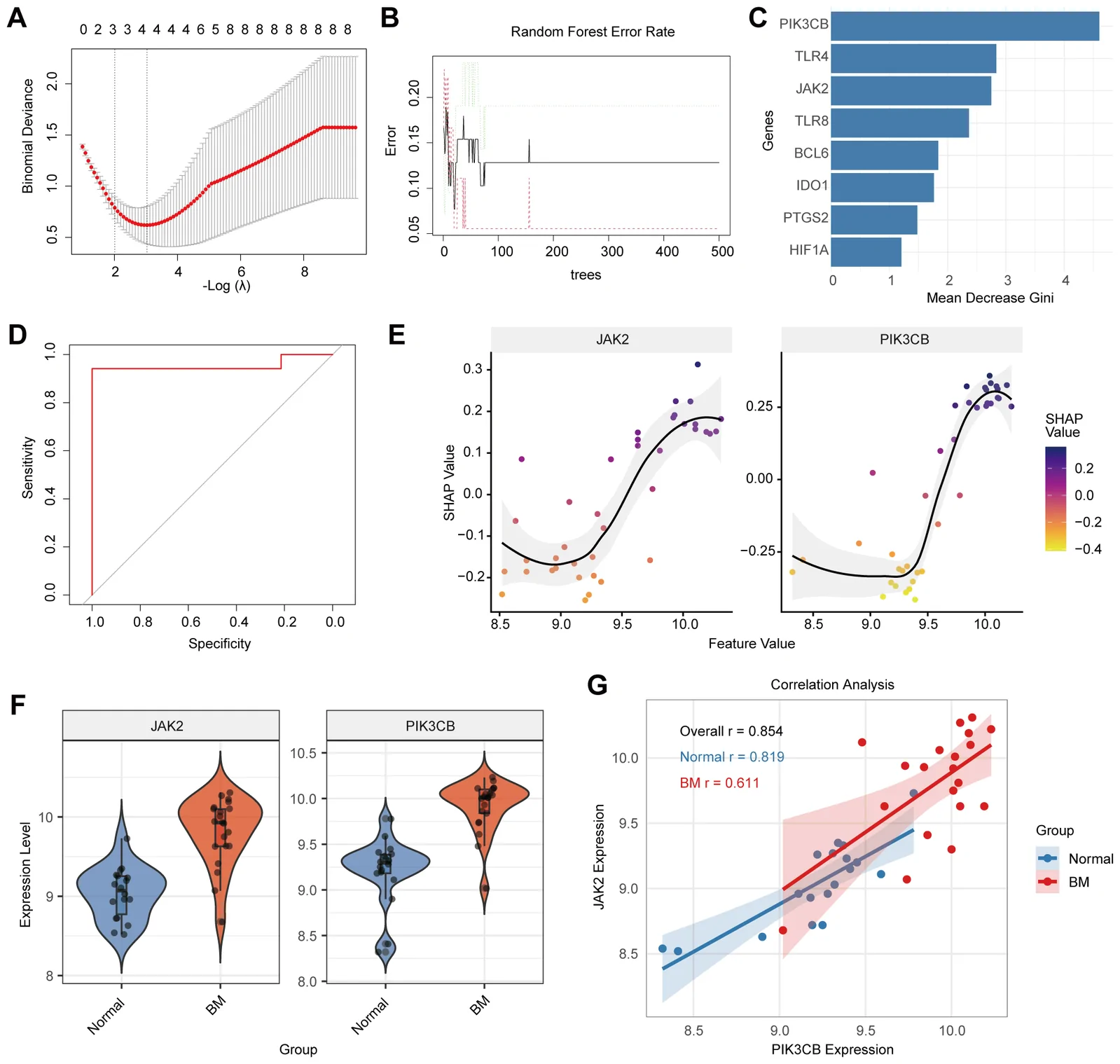
Machine learning-based screening and validation of core genes. **(A)** Cross-validation curve for LASSO regression, illustrating the selection of the optimal penalty parameter λ through 5-fold cross-validation. The x-axis represents log(λ), and the y-axis represents binomial deviance. The red dashed lines indicate the positions of lambda.min and lambda.1se, with the vertical dashed line corresponding to lambda.1se, at which feature genes with non-zero coefficients were selected. **(B)** Out-of-bag (OOB) error plot of the random forest model, showing the trend of OOB error as the number of decision trees (ntree) increases. The error stabilized when the number of decision trees reached approximately 500. **(C)** Random forest feature importance ranking. The importance of each gene was calculated based on the Gini coefficient (Mean Decrease Gini) and ranked in descending order. The red dashed line represents the median importance; genes above this line were included in subsequent intersection analysis. **(D)** Receiver operating characteristic (ROC) curve for diagnostic performance validation. A random forest model was constructed based on the selected key feature genes (PIK3CB and JAK2), and the ROC curve was generated using 5-fold cross-validation. **(E)** SHAP feature contribution analysis. The SHAP method was used to interpret the final model, showing the contribution of PIK3CB and JAK2 to the prediction outcome. Each dot represents a sample, with color indicating gene expression level (purple: high expression; yellow: low expression). The x-axis represents the SHAP value (positive values push the prediction toward case; negative values push the prediction toward control), revealing the direction and strength of influence for each gene. **(F)** Boxplot showing the expression levels of key genes (PIK3CB and JAK2) in the meningitis (BM) and normal control groups. **(G)** Scatter plot showing the correlation between PIK3CB and JAK2 expression levels.

To systematically screen core genes, topological analysis of the network was conducted using five different algorithms (Degree, EPC, MCC, MNC, and Radiality) with the cytoHubba plugin (**Figures 3A–E**). Each algorithm screened the top 10 genes, and the intersections of the core genes obtained by the five algorithms were collected, yielding a set of stable intersecting core genes (**Figure 3F**). To further identify key diagnostic markers from the core genes screened by the PPI network, two machine learning algorithms, LASSO regression and random forest, were comprehensively applied for feature selection. Using the expression data of the intersecting core genes common to the five algorithms as input features and sample grouping as the response variable, LASSO regression combined with 5-fold cross-validation was applied to select feature genes with non-zero coefficients (15). Simultaneously, based on the feature importance scores (Mean Decrease Gini) from the random forest model, genes with importance above the median were selected. The intersection of the genes selected by these two methods revealed two key feature genes: PIK3CB and JAK2 (**Figures 4A–C**).

To evaluate the diagnostic performance of these two genes, the random forest model was validated using 5-fold cross-validation. This model demonstrated good predictive performance, with an average AUC value of 0.898 (**Figure 4D**). SHAP interpretability analysis further quantified the contribution of PIK3CB and JAK2 to model predictions, confirming them as key features distinguishing patients with TBM from normal controls (**Figure 4E**). The boxplot results demonstrated that the expression levels of both JAK2 and PIK3CB were significantly upregulated in the disease group (**Figure 4F**). Moreover, a significant positive correlation between JAK2 and PIK3CB expression was observed in both the normal and disease groups (**Figure 4G**).

### Immune microenvironment characterization

**Figure 5:**
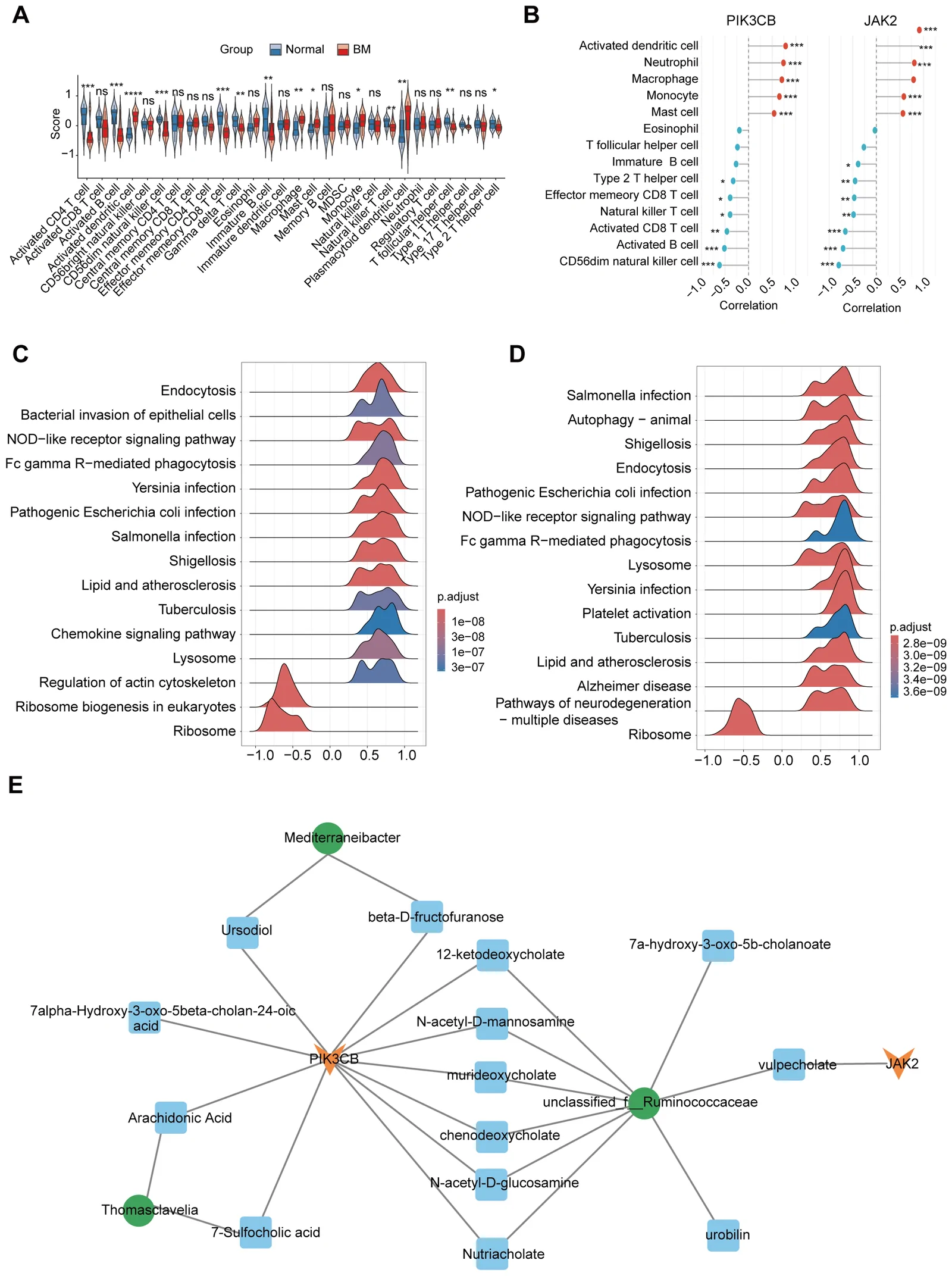
Analysis of immune microenvironment characteristics. **(A)** Based on the GSE40586 dataset, the ssGSEA algorithm was used to calculate immune cell enrichment scores for each sample, comparing differences between the BM group (red) and the Normal group (blue). **(B)** Heatmap/dot plot showing the correlation between core genes and immune cells. Pearson correlation analysis results between PIK3CB and JAK2 expression levels and differential immune cell enrichment scores are displayed. **(C)** Ridge plot of single-gene GSEA for JAK2. Gene sets were ranked based on Spearman correlation coefficients between JAK2 expression and all other genes, and enriched KEGG pathways were identified by GSEA. Red peaks represent pathways positively correlated with JAK2 expression, while blue peaks represent negatively correlated pathways. **(D)** Ridge plot of single-gene GSEA for PIK3CB. GSEA enrichment results based on PIK3CB expression correlation. **(E)** Microbiota-metabolite-gene interaction network. A network was constructed by integrating significantly upregulated gut microbiota (green circular nodes) in TBM patients, metabolites derived from the gutMGene database (blue squares), and core genes (large orange/pink nodes, PIK3CB and JAK2). Connecting lines represent known associations between microbiota and metabolites or between metabolites and genes.

To explore the immune microenvironment characteristics, ssGSEA immune infiltration analysis based on the GSE40586 dataset, comparing the enrichment scores of various immune cells between the BM group (red) and the Normal group (blue) was conducted (**Figure 5A**). The violin plot showed that the enrichment scores of several pro-inflammatory and innate immune cell types, including activated dendritic cells, neutrophils, macrophages, monocytes, mast cells, some activated CD4/CD8 T cell subsets, and type 1/2 helper T cells (Th1/Th2), were significantly higher in the BM group compared with the Normal group. Conversely, the enrichment scores of some memory T cells, regulatory T cells (Tregs), and certain natural killer cell subsets were significantly lower in the BM group. These results indicate that patients with TBM exhibit a significant increase in pro-inflammatory/innate immune cell infiltration, concurrent with and an imbalance in adaptive immunity. Furthermore, we analyzed their correlation with immune cell infiltration (**Figure 5B**). The left panel shows the results of Pearson correlation analysis between gene expression and immune cell enrichment scores. PIK3CB expression showed a significant positive correlation with the infiltration levels of several pro-inflammatory immune cells. JAK2 showed a similar expression pattern, with even stronger positive correlations with pro-inflammatory cells. These results suggest that high expression of PIK3CB and JAK2 is closely associated with the pro-inflammatory immune microenvironment in BM and may be key genes driving the inflammatory response.

To further investigate the potential biological functions of PIK3CB and JAK2 in TBM, single-gene GSEA analysis was conducted based on their expression correlation, displaying the enrichment results using ridge plots (**Figures 5C, D**). The results revealed that gene sets positively correlated with the expression of PIK3CB and JAK2 (red peaks) were significantly enriched in multiple pathways related to infection and immune inflammation. Common significantly positively enriched pathways included Yersinia infection, Salmonella infection, shigellosis, pathogenic Escherichia coli infection, NOD-like receptor signaling, Fc gamma R-mediated phagocytosis, autophagy, lysosome, and tuberculosis itself. In contrast, gene sets negatively correlated with PIK3CB and JAK2 expression (blue peaks) were predominantly enriched in protein synthesis-related pathways, such as ribosome and ribosome biogenesis. These results consistently indicate that high expression of PIK3CB and JAK2 is closely coupled with processes such as bacterial infection response, phagocytosis, autophagy regulation, and lysosomal function, which are all highly consistent with the pathophysiology of tuberculosis.

To explore the potential regulatory effect of the upstream gut microbiota and their metabolites on the core genes, we integrated the differential microbiota, metabolite information from the gutMGene database, and the core gene targets to construct a microbiota-metabolite-gene interaction network (**Figure 5E**). In this network, PIK3CB and JAK2 served as core gene nodes (large orange/pink nodes), connected to multiple metabolite nodes (blue squares). These metabolites mainly included bile acids (e.g., ursodeoxycholate, chenodeoxycholate), lipids (e.g., arachidonic acid), and amino sugars (e.g., N-acetyl-D-glucosamine). Further tracing revealed that these metabolites were associated with several gut bacterial groups found to be significantly upregulated in patients with TBM (green circular nodes), predominantly including Mediterraneibacter, unclassified f_Ruminococcaceae, and Thomasclavelia. This network reveals a potential “gut-brain axis” regulatory pathway, indicating that specific gut bacteria upregulated in patients with TBM (such as members of the Ruminococcaceae family) may generate specific metabolites (such as bile acids and fatty acid derivatives), which in turn regulate the expression of the host key immune genes PIK3CB and JAK2, thereby participating in the pathological process of TBM.

### Construction of the microbiota-metabolite-gene interaction network

**Figure 6:**
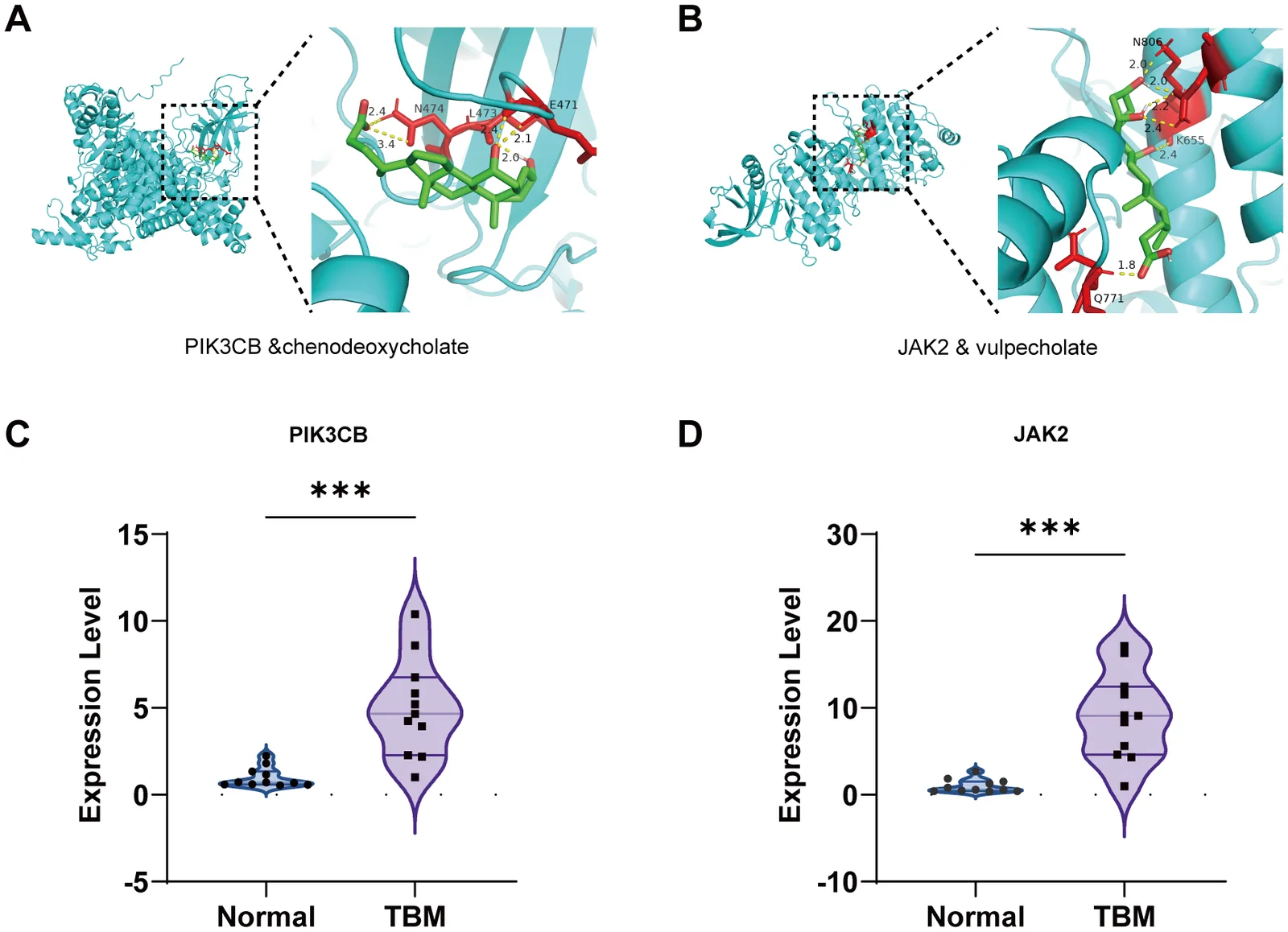
Construction of the microbiota-metabolite-gene interaction network. **(A, B)** Representative metabolites associated with core genes were subjected to molecular docking with the PIK3CB and JAK2 proteins. **(C, D)** Relative expression levels of PIK3CB and JAK2 mRNA were detected by RT-qPCR in PBMCs from TBM patients and healthy controls (n = 11). * indicates statistical significance between groups, ***P < 0.001.

To investigate whether the upstream differential microbiota-related metabolites may directly act on the core genes PIK3CB and JAK2 from a structural biology perspective, molecular docking studies were performed. Based on the microbiota-metabolite-gene network constructed in **Figure 6A**, metabolites associated with core genes were identified and molecular docking with PIK3CB and JAK2 proteins were separately identified. The binding energy (kcal/mol) was calculated to assess their potential binding affinity, for which a lower binding energy indicates a greater likelihood of binding and more stable interaction. The results showed that PIK3CB exhibited varying degrees of binding ability with multiple metabolites. Among these, chenodeoxycholate and murideoxycholate displayed the strongest binding affinity, with low binding energies of -7.76 kcal/mol and -7.59 kcal/mol, respectively (**Figure 6A**; **Supplementary Figure 2**). Additionally, other bile acid derivatives, such as 7a_hydroxy_3_oxo_5b_cholanoate (-6.59 kcal/mol), nutriacholate (-6.53 kcal/mol), ursodiol (-6.33 kcal/mol), vulpecholate (-6.24 kcal/mol), 7-sulfocholic acid (-6.03 kcal/mol), and 12-ketodeoxycholate (-5.81 kcal/mol), also showed strong binding potentials (binding energy < -5.0 kcal/mol). In contrast, arachidonic acid (-2.79 kcal/mol) and amino sugar metabolites such as N_acetyl_D_glucosamine (-3.59 kcal/mol) and N-acetyl-D-mannosamine (-3.05 kcal/mol) had higher binding energies indicating relatively weaker affinities. Molecular docking results for JAK2 showed that vulpecholate had an extremely strong binding affinity for the JAK2 protein, with a binding energy of -6.81 kcal/mol (**Figure 6B**). Comprehensive analysis of the molecular docking results revealed the following: Bile acid metabolites were the core ligands; various bile acid derivatives (particularly chenodeoxycholate, murideoxycholate, and vulpecholate) exhibited low binding energies (< -6.0 kcal/mol) with both PIK3CB and JAK2, indicating that these molecules produced by gut microbial metabolism may act as be key effector molecules regulating core gene activity. Furthermore, selectivity between metabolites and targets was found; specifically, vulpecholate showed a strong binding ability to both PIK3CB (-6.24 kcal/mol) and JAK2 (-6.81 kcal/mol), potentially acting as a broad-spectrum effector molecule, while chenodeoxycholate and murideoxycholate showed stronger selectivity for PIK3CB. The binding energies for most interactions were all well below the -5.0 kcal/mol threshold, indicating that these interactions are not random and have potential biological functional significance. To validate the expression of the core genes identified by bioinformatics analysis, the relative mRNA expression levels of PIK3CB and JAK2 in PBMCs from TBM patients and healthy controls were assessed by RT-qPCR (Biological replicates n = 11, with 3 technical replicates per sample). As shown in **Figure 6B**, compared with the healthy control group, the mRNA expression levels of both PIK3CB and JAK2 were significantly elevated in the TBM group, with statistical significance (**Figures 6C, D**). These experimental results were highly consistent with the differential expression trends observed in the GSE40586 transcriptomic dataset, further confirming that PIK3CB and JAK2 are significantly upregulated in the peripheral blood of patients with TBM. This finding suggests that PIK3CB and JAK2 may serve as potential key molecular markers involved in the pathogenesis of TBM.

### CDCA promotes THP-1 macrophage activation via the PIK3CB/JAK2

**Figure 7:**
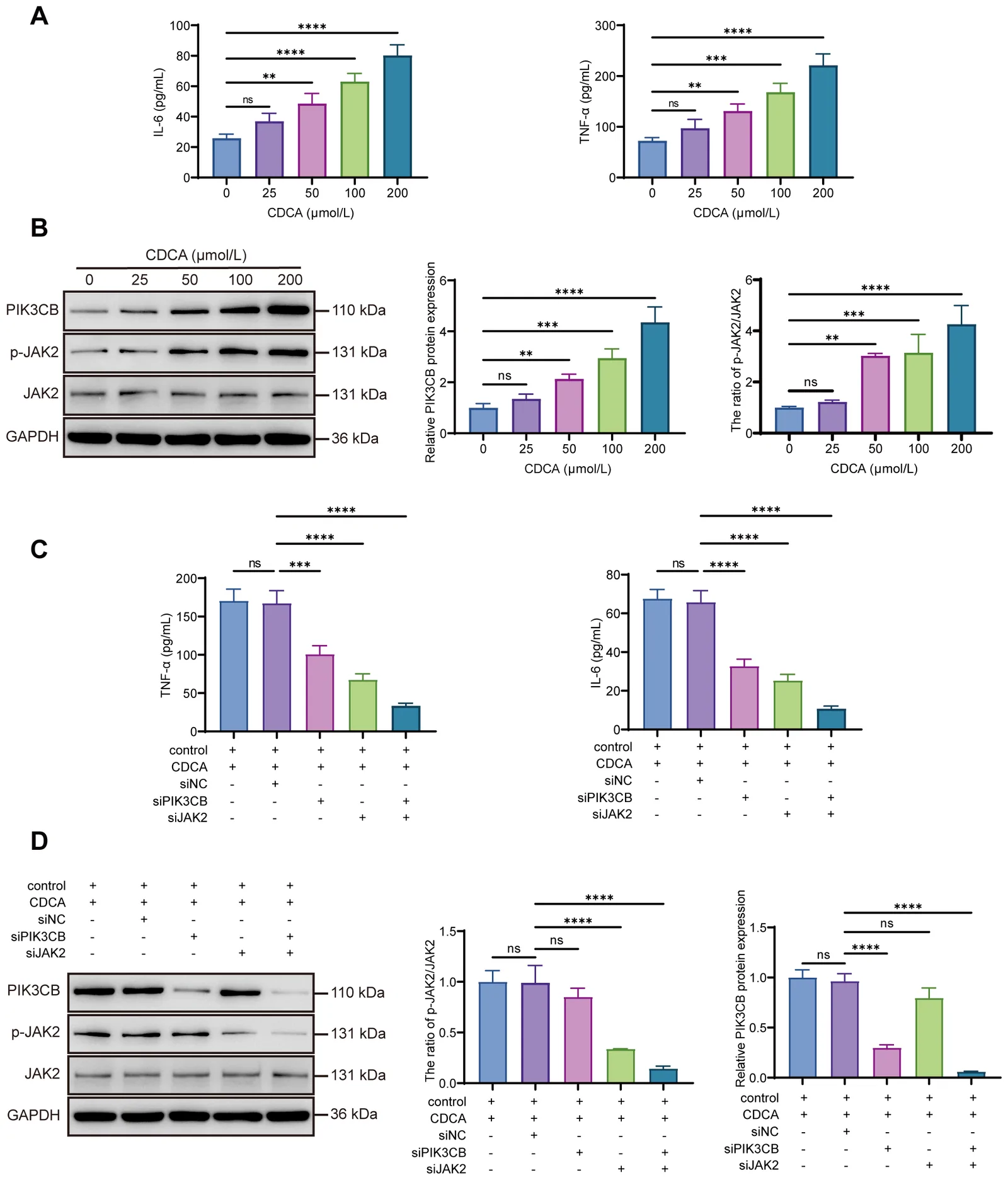
CDCA mediates pro-inflammatory activation of macrophages via the PIK3CB/JAK2 pathway. **(A)** THP-1 macrophages were treated with gradient concentrations of CDCA for 24 h. The secretion levels of IL-6 and TNF-α in cell supernatant were detected by ELISA to verify the dose-dependent pro-inflammatory effect of CDCA. **(B)** After treatment with indicated concentrations of CDCA, protein expression levels of PIK3CB, total JAK2 and phosphorylated JAK2 (p-JAK2) were detected by Western blot. The right panels show quantitative densitometry analysis of relative PIK3CB protein level and the ratio of p-JAK2 to total JAK2, respectively. **(C)** Macrophages were transfected with negative control siRNA (siNC), PIK3CB-targeting siRNA or JAK2-targeting siRNA, followed by CDCA treatment. The secretion levels of TNF-α and IL-6 in cell supernatant were detected by ELISA to verify the reversal effect of target gene silencing on CDCA-induced inflammation. **(D)** Western blot results of the corresponding groups. The right panels show quantitative densitometry analysis of the p-JAK2/total JAK2 ratio and relative PIK3CB protein level, respectively, verifying the knockdown efficiency of siRNAs and their inhibitory effect on pathway activation. * indicates statistical significance between groups, **P < 0.01, ***P < 0.001, ****P < 0.0001.

The pro-inflammatory regulatory effects of chenodeoxycholic acid (CDCA) were validated in a PMA-induced THP-1 macrophage model through dose-gradient treatment and siRNA gene silencing rescue experiments. The results showed that higher concentrations of CDCA affected the viability of THP-1 cells, with cell viability decreasing to approximately 50% at 100 μM (**Supplementary Figure 3A**). CDCA promotes the secretion of pro-inflammatory factors such as IL-6 and TNF-α by macrophages in a dose-dependent manner, while simultaneously upregulating total PIK3CB protein expression and enhancing the phosphorylation and activation of JAK2; the expression levels of both increased progressively with rising CDCA concentrations (**Figures 7A, B**; **Supplementary Figure 3B**). Specific knockdown of either PIK3CB or JAK2 significantly attenuated CDCA-induced secretion of inflammatory factors, and dual silencing of both PIK3CB and JAK2 almost completely blocked the pro-inflammatory effects of CDCA, with a significantly stronger effect than single-gene silencing (**Supplementary Figure 4**; **Figure 7C**). At the same time, silencing of either PIK3CB or JAK2 did not affect the expression of the other target (**Figure 7D**). These results confirm that PIK3CB and JAK2 are essential downstream targets for CDCA-mediated inflammatory responses in macrophages, and that both genes synergistically participate in the pro-inflammatory regulation of innate immune cells by bile acids. This further validates the rationale behind the aforementioned metabolite-gene interaction target screening.

## Discussion

The pathogenesis of TBM, the most lethal form of extrapulmonary tuberculosis, remains incompletely understood. In recent years, research on the regulating role the gut microbiota exerts on central nervous system function via the “gut-brain axis” has provided new perspectives for understanding the pathological processes of TBM. By integrating 16S rRNA sequencing, metabolite target prediction, transcriptomic validation, and molecular docking, this study systematically revealed the characteristics of gut microbiota dysbiosis in patients with TBM, as well as the potential mechanisms by which they regulate host immune gene expression through metabolites, thus providing experimental evidence to promote a deeper understanding of TBM pathogenesis. This study identified significant alterations in gut microbiota structure in patients with TBM, characterized by a clear separation in β-diversity from healthy controls and a significant reduction in OTU richness. At the compositional level, the relative abundances of Fusicatenibacter and Faecalibacterium were found to be significantly reduced in the TBM group, while Thomasclavelia, unclassified f_Ruminococcaceae, and Mediterraneibacter were all significantly elevated. This finding is consistent with prior studies. For example, Li et al. also reported a reduction in the Firmicutes phylum in the gut microbiota of patients with TBM. However, there were also some differences. For example, Li et al. found a significant increase in Escherichia-Shigella, whereas the elevated genera in this study were mainly Thomasclavelia and some members of the Ruminococcaceae family. These differences may be related to factors such as geographical region, antibiotic exposure, disease severity, or detection platform (16). Consistent with most published studies on tuberculosis and TBM, this study also observed significant gut microbiota dysbiosis in TBM patients, characterized by reduced α-diversity and decreased abundance of butyrate-producing probiotic genera such as Faecalibacterium. This common feature suggests that impaired gut anti-inflammatory capacity and microbiota imbalance are both universal characteristics of Mycobacterium tuberculosis infection. Consistent with Li et al.’s report of significant enrichment of the Escherichia-Shigella genus in the intestines of TBM patients, this genus was also significantly enriched in our study cohort, albeit in the opposite direction. A study by Ye et al. involving 69 untreated pulmonary tuberculosis (PTB) patients confirmed a significant decrease in the abundance of genera such as Faecalibacterium and Bifidobacterium (17). This is consistent with our findings. We attribute this discrepancy to key factors such as treatment status, geographic baseline, and timing of sampling. Drugs can cause profound and rapid disruptions to the gut microbiome. Some patients included in this study had already received standard anti-tuberculosis treatment for more than 72 hours at the time of sample collection; this may have resulted in the peak of systemic inflammation having already passed. The timing of sampling may also explain why no significant enrichment of specific pathogens was observed. Furthermore, patients in the HC group may have had a history of other diseases, and this difference may be the primary reason for the observed disparity in E. coli and Shigella abundances. Additionally, geographic location, dietary patterns, and living environments all have significant effects on the baseline gut microbiome of populations (18, 19). Notably, through urinary metabolomics, Isaiah et al. identified eight microbiota-related metabolite markers in pediatric patients with TBM, further confirming the presence of gut microbiota metabolic disturbances in this population (20). The above results reveal that patients with TBM exhibit a gut microbiota structural disorder characterized by a reduction in beneficial bacteria and an increase in potential pathogenic bacteria, thereby providing new clues for the role of the “gut-brain axis” in TBM pathogenesis, and suggesting potential avenues for future adjunctive interventions.

To further elucidate how the gut microbiota participates in the TBM pathological process, we constructed a “microbiota-metabolite-gene” interaction network using the bacterial meningitis dataset GSE40586. This network showed that upregulated microbiota (Thomasclavelia, unclassified f_Ruminococcaceae, Mediterraneibacter) may act on the host core immune genes PIK3CB and JAK2 by generating bile acid metabolites (such as chenodeoxycholate, vulpecholate, etc.). Molecular docking results further validated the strong binding affinity of these metabolites with the target proteins (binding energy < -6.0 kcal/mol). Importantly, the gut-brain axis involves a bidirectional regulatory mechanism; the central nervous system infection can reciprocally influence gut microbiota composition through activation of the hypothalamic-pituitary-adrenal axis and autonomic nervous system regulation, thereby forming a positive feedback loop (21). Isaiah et al. previously proposed the “top-down” concept, suggesting that chronic central nervous system infection can induce gut dysbiosis via action on the neuroimmune-endocrine network (22, 23), which in turn can exacerbate neuroinflammation through metabolite feedback. Subsequent urinary metabolomics investigation by the same team further confirmed the existence of significant alterations in eight microbiota-related metabolites in pediatric patients with TBM, thus providing direct evidence for the existence of this bidirectional loop (20). The microbiota-metabolite-gene regulatory axis identified in the present study may represent a crucial molecular basis for this bidirectional loop. Although the specific role of PIK3CB (phosphatidylinositol-3-kinase catalytic subunit beta) itself in meningitis has not been reported, PIK3CB is an important member of the PI3K family which has been extensively studied in meningitis, as PI3K is primarily involved in the invasion of the blood-brain barrier by *Neisseria meningitidis*. This kinase is activated by the pathogen, mediating cytoskeletal rearrangement and bacterial internalization, and regulating excessive inflammatory responses (24, 25). JAK2, a central hub for cytokine signaling, drives the inflammatory response of microglia and immune cells by activating the STAT pathway, and inhibition of this pathway can alleviate brain tissue damage (26). These two genes reveal meningitis mechanisms from the perspectives of pathogen invasion and host immune response, respectively, although research on them as therapeutic targets remains in its early stages. Our experiments also only confirmed that PIK3CB and JAK2 are effective targets for CDCA and can regulate the pro-inflammatory effect of macrophages.

This study has several innovations. First, in terms of methodology, this study combined 16S rRNA sequencing, gutMGene database mining, SuperPred target prediction, and GEO transcriptomic data to construct a multi-omics integration analysis strategy, thus providing a systematic solution to reveal the “microbiota-metabolite-gene” cross-domain regulation. Second, in terms of elucidating mechanisms, this study not only identified differential microbiota associated with TBM but also validated, through molecular docking analysis, the potential for direct binding between gut microbial metabolites (particularly bile acids) and the core genes PIK3CB and JAK2. This represents a relatively innovative study that systematically validates the bile acid–PIK3CB/JAK2 inflammatory regulatory axis in tuberculous meningitis by combining machine learning screening with molecular docking simulation techniques. Third, at the clinical application level, machine learning algorithms (LASSO regression and random forest) were applied to screen PIK3CB and JAK2 as diagnostic feature genes, and their correlation with the pro-inflammatory immune microenvironment was revealed through immune infiltration analysis, thereby offering new directions for biomarker development and therapeutic target selection for TBM.

Nevertheless, this study also has limitations. Firstly, the sample size of participants used for 16S rRNA sequencing was small (n=11/group), which might affect the statistical power for detecting differences in the microbial community. Therefore, a larger sample size is required for verification. When conducting extensive sample verification, it is necessary to conduct power analysis or effect size estimation to enhance the robustness of the research results. Second, we used OTU-based clustering at 97% similarity, which, while widely employed in microbiome research, may have lower taxonomic resolution compared to amplicon sequence variant (ASV)-based methods. Future studies employing ASV-based approaches may provide finer taxonomic resolution. At the same time, some of the machine learning models have only been validated through internal cross-validation and have not been validated for general applicability in an external, independent dataset. The metabolite-microbiota associations in the gutMGene database were primarily derived from experimental validation, meaning they may be subject to reporting bias, and some metabolites associated with differential microbiota may not have been fully covered. Third, the molecular docking results only indicate the binding potential of metabolites to target proteins; their actual regulatory activity and biological functions require validation through *in vivo* animal model experiments. Fourth, the GSE40586 dataset is derived from patients with bacterial meningitis, although it is different from the cause of TBM, both may share similar host inflammatory response pathways during the acute phase, such as Toll-like receptor signaling pathways, NOD-like receptor pathways, and inflammatory cytokine storms, which provides a biological basis for the cross-disease screening in this study. In the future, we plan to expand the clinical sample size and collect matched gut microbiome, transcriptomic, and metabolomic data from this same TBM cohort. We will use established multi-omics integration algorithms, such as DIABLO, to construct a formal integrated model and more systematically analyze the brain-gut regulation of TBM.

Based on these limitations, future research should therefore focus on the following directions: First, establishing TBM animal models and using fecal microbiota transplantation experiments to validate the regulatory effects of specific microbiota (such as Mediterraneibacter) and their metabolites (such as chenodeoxycholate) on PIK3CB and JAK2 expression and BBB function, or further validated in an independent TBM clinical cerebrospinal fluid dataset. Second, conducting prospective cohort studies to dynamically monitor the trajectories of changes in gut microbiota, metabolite profiles, and immune gene expression during treatment in patients with TBM, and assessing their associations with clinical outcomes. Third, exploring microbiota-based intervention strategies, such as supplementation with butyrate-producing bacteria or using bile acid metabolism modulators, to open new avenues for adjunctive therapy in TBM.

## Data Availability

The datasets used and/or analyzed during the current study are available from the corresponding author on reasonable request.
