# Supplementary material for: Integrating metagenomics, transcriptomics, and molecular docking to reveal core gene biomarkers and gut microbiota regulatory mechanisms in tuberculous meningitis
Source: Front Immunol. 2026 Sep 9;17:1844914. doi: 10.3389/fimmu.2026.1844914 (PMC13597347; doi:10.3389/fimmu.2026.1844914)
Supplement: Supplementary file 1 [file SupplementaryFile1.docx]

**Table S1 Primer Sequences**

| **Gene** | **Species** | **SS Sequence** | **AS Sequence** |
| --- | --- | --- | --- |
| PIK3CB | Human | ATGAAGCCTTTGTGGCTGGT | CTTCAATGAGGCCAGAGCGA |
| JAK2 | Human | TGCCGGTATGACCCTCTACA | ACCAGCACTGTAGCACACTC |
| GAPDH | Human | GGAGCGAGATCCCTCCAAAAT | GGCTGTTGTCATACTTCTCATGG |

**Table S2 Knockdown primer sequence**

| **Gene** | **Species** | **SS Sequence** | **AS Sequence** |
| --- | --- | --- | --- |
| PIK3CB-1 | Human | GAGAACUAAAUUUGAAUUAAG | UAAUUCAAAUUUAGUUCUCAA |
| PIK3CB-2 | Human | CUUUCUCAUUUUUAAAACAUG | UGUUUUAAAAAUGAGAAAGUU |
| PIK3CB-3 | Human | CAAAUCCAUAUACUGAAAAUG | UUUUCAGUAUAUGGAUUUGUU |
| JAK2-1 | Human | CAAGAUGUGAACUGUUUCUCU | AGAAACAGUUCACAUCUUGUU |
| JAK2-2 | Human | CUCUUCUAUAUAUCAGAAUGG | AUUCUGAUAUAUAGAAGAGGU |
| JAK2-3 | Human | CUUUAAUGAGUGAAACAGAAA | UCUGUUUCACUCAUUAAAGCA |
| NC | Human | UUCUCCGAACGUGUCACGUTT | ACGUGACACGUUCGGAGAATT |

**Figure S1 Validation of Sequencing Saturation for Gut Microbiome Alpha Diversity and Analysis of Intergroup Differences**

(A) Dilution curves for the number of observed species (Sobs); (B) Dilution curves for the Shannon index. The red curve represents the healthy control group (HC), and the blue curve represents the tuberculous meningitis group (TBM);

(C) The Sobs index reflects microbial species richness; (D) The Shannon index comprehensively reflects species richness and evenness; (E) The Simpson index reflects community species dominance and evenness. Scatterplots represent individual samples. ***P<0.001.

**Figure S2 Molecular Docking Site Diagram**

**Figure S3 Effects of CDCA on THP-1 Cell Viability and on the PIK3CB and JAK2 Targets**

A: CCK8 assay to assess the effect of different concentrations of CDCA on cell viability (n=3); B: mRNA levels of PIK3CB and JAK2 targets at different CDCA concentrations (n=3). *P<0.05, **P<0.01, ***P<0.001, ****P<0.0001, ns: No significant difference.

**Figure S4 Validation of knockdown efficiency of PIK3CB and JAK2 specific siRNAs**

A: Relative mRNA expression of PIK3CB after transfection with PIK3CB-targeting siRNAs (n=3); B: Relative mRNA expression of JAK2 after transfection with JAK2-targeting siRNAs (n=3). ***P<0.001, ****P<0.0001.
